# Estradiol Drives the Anorexigenic Activity of Proopiomelanocortin Neurons in Female Mice

**DOI:** 10.1523/ENEURO.0103-18.2018

**Published:** 2018-10-10

**Authors:** Todd L. Stincic, Pasha Grachev, Martha A. Bosch, Oline K. Rønnekleiv, Martin J. Kelly

**Affiliations:** 1 Department of Physiology and Pharmacology, Oregon Health and Science University, Portland, OR 97239; 2 Division of Neuroscience, Oregon National Primate Center, Oregon Health and Science University, Beaverton, OR 97006

**Keywords:** estrogen, obesity, sex differences, STX

## Abstract

Energy balance is regulated by anorexigenic proopiomelanocortin (POMC) and orexigenic neuropeptide Y/agouti-related peptide (NPY/AgRP) neurons of the hypothalamic arcuate nucleus. POMC neurons make extensive projections and are thought to release both amino acid and peptide neurotransmitters. However, whether they communicate directly with NPY/AgRP neurons is debated. Initially, using single-cell RT-PCR, we determined that mouse POMC^eGFP^ neurons express *Slc17a6* (*Vglut2*) and *Slc18a2* (*Vmat2*), but not *Slc31a1* (*Vgat*) mRNA, suggesting glutamate and non-canonical GABA release. Quantitative (q)RT-PCR of POMC^eGFP^ cells revealed that *Vglut2* and *Vmat2* expression was significantly increased in E2- versus oil-treated, ovariectomized (OVX) female mice. Since 17β-estradiol (E2) is anorexigenic, we hypothesized that an underlying mechanism is enhancement of POMC signaling. Therefore, we optogenetically stimulated POMC neurons in hypothalamic slices to examine evoked release of neurotransmitters onto NPY/AgRP neurons. Using brief light pulses, we primarily observed glutamatergic currents and, based on the paired pulse ratio (PPR), determined that release probability was higher in E2- versus oil-treated, OVX female, congruent with increased *Vlgut2* expression. Moreover, bath perfusion of the Gq-coupled membrane estrogen receptor (ER) agonist STX recapitulated the effects of E2 treatment. In addition, high-frequency (20 Hz) stimulation generated a slow outward current that reversed near E_k+_ and was antagonized by naloxone, indicative of β-endorphin release. Furthermore, individual NPY/AgRP neurons were found to express *Oprm1*, the transcript for μ-opioid receptor, and DAMGO, a selective agonist, elicited an outward current. Therefore, POMC excitability and neurotransmission are enhanced by E2, which would facilitate decreased food consumption through marked inhibition of NPY/AgRP neurons.

## Significance Statement

Proopiomelanocortin (POMC) neurons of the hypothalamic arcuate nucleus sense the energy state of an animal and regulate satiety to maintain homeostasis. Neuropeptide Y/agouti-related peptide (NPY/AgRP) neurons also participate in energy balance, but instead drive hunger. Disruptions in this circuit can promote the development of obesity, which frequently occurs during menopause due to the loss of estrogens. The primary female sex hormone, 17β-estradiol (E2) exerts an anorexigenic effect, decreasing food intake and increasing activity. These behavioral changes are mediated, in part, through inhibition of NPY/AgRP and potentiation of POMC signaling. For the first time, we report that POMC neurons provide direct input to NPY/AgRP neurons primarily through glutamate and β-endorphin release. Furthermore, E2 enhances POMC neurotransmission to inhibit NPY/AgRP neurons.

## Introduction

17β-estradiol (E2) is involved in the regulation of appetite, energy expenditure, body weight and adipose tissue deposition/distribution in females (Milewicz et al., 2000; Geary, 2001). During hypoestrogenic states (e.g., menopause) women tend to decrease activity and gain body fat with the increase in adiposity correlated to the decline in endogenous estrogens (Carr, 2003; Augoulea et al., 2005; Jasienska et al., 2005). In rodent models, ovariectomy (OVX) induces an increase in food intake and a decrease in ambulatory and wheel running activities, all of which are reversed with E2 replacement (Colvin and Sawyer, 1969; Ahdieh and Wade, 1982; Shimomura et al., 1990; Asarian and Geary, 2002; Qiu et al., 2006). The effect of E2 on ingestive behavior is mediated both by attenuating hunger and enhancing satiety. Within the arcuate nucleus of the hypothalamus (ARH), orexigenic neuropeptide Y/agouti-related peptide (NPY/AgRP) and anorexigenic proopiomelanocortin (POMC) neurons are critical to energy balance. Optogenetic activation of NPY/AgRP neurons drives hunger, whereas similar stimulation of POMC neurons decreases consumption (Aponte et al., 2011). These populations maintain homeostasis by monitoring and responding to circulating indicators of energy state (e.g., blood glucose, leptin, insulin, and ghrelin), which is possible due to their proximity to the 3rd ventricle and median eminence, a circumventricular organ (Broadwell et al., 1983; Norsted et al., 2008).

The divergent actions of E2 and other hormones (Elias et al., 1999; Qiu et al., 2014) on NPY/AgRP and POMC neurons are well established, and these ARH neurons clearly act in an antagonistic fashion on downstream targets, as AgRP is an endogenous antagonist of melanocortin receptors (Ollmann et al., 1997). Yet, the existence of direct connections between these two populations have been debated. Only a single study has specifically probed connectivity, finding NPY/AgRP to POMC inputs but not the reciprocal connection (Atasoy et al., 2012). However, it must be noted that low-frequency/constant stimulation was used, the internal solution was biased to measure GABAergic responses, and the actual number of cells recorded was low. The first distinction is important, as high-frequency (20Hz) optogenetic stimulation can release glutamate that is excitatory on POMC and inhibitory on NPY/AgRP neurons through differential expression of postsynaptic metabotropic glutamate receptors (Nestor et al., 2016). Also, while NPY/AgRP neurons receive GABAergic input from multiple sources, one of which may be POMC neurons, POMC release of GABA is another point of contention as they do not express *Vgat* (Ovesjö et al., 2001; Hentges et al., 2004). Yet, optogenetic stimulation of POMC^Cre^::ChR2 neurons appears to evoke GABA and glutamate release, however either the number of postsynaptic neurons tested was small or their identity unknown (Atasoy et al., 2008; Dicken et al., 2012). In addition, high-frequency stimulation has been shown to release neuropeptides (Piñol et al., 2012; Kempadoo et al., 2013; Walsh et al., 2014; Apergis-Schoute et al., 2015; Qiu et al., 2016), and β-endorphin input from POMC neurons could dampen NPY/AgRP neuronal activity in fed states (Yang et al., 2011). β-Endorphin binds to μ-opioid receptors, which activates G protein-coupled inwardly rectifying K^+^ (GIRK) channels to hyperpolarize ARH neurons (Kelly et al., 1990; Slugg et al., 2000; Pennock and Hentges, 2011). If peptide release is the predominant form of POMC to NPY/AgRP neurotransmission then previous studies could have easily overlooked this putative synapse. Also, perhaps the steroid or energy state of the animal is critical to observe certain ARH interactions, and we already know NPY/AgRP projections are highly plastic (Pinto et al., 2004), a trait potentially shared by neighboring POMC neurons.

Although there has been a concerted effort to elucidate the effects of ionotropic and metabotropic receptor agonists on POMC and NPY/AgRP neurons over the past twenty-five years, the postsynaptic effects of evoked amino acid neurotransmitter release is only just emerging with the advent of optogenetics (Atasoy et al., 2008; Dicken et al., 2012; Rau and Hentges, 2017). Furthermore, nothing is known about the evoked release of neuropeptides or sex differences in transmission in relation to this homeostatic neurocircuit. Therefore, in the present study we targeted NPY/AgRP neurons using voltage clamp to record postsynaptic responses following low-frequency and high-frequency optogenetic stimulation of POMC^Cre::^ChR2 neurons in both male and female mice to address these questions.

## Materials and Methods

### Animals

Both male and female mice were used throughout this experiment. All animal procedures were conducted according to the National Institutes of Health Guide for the Care and Use of Laboratory Animals and with approval from the Oregon Health and Science University Animal Care and Use Committee. Animals were bred and housed in a specific pathogen-free area.

POMC^Cre^ mice originally obtained from The Jackson Laboratories (RRID:IMSR_JAX:005965; Balthasar et al., 2004) were crossed with either Ai32 (RRID:IMSR_JAX:012569; Madisen et al., 2012) or NPY^hrGFP^ transgenic mice (RRID:IMSR_JAX:006417; van den Pol et al., 2009). POMC^eGFP^ mice (Cowley et al., 2001) were used in the single-cell RT-PCR experiments. Ai32 mice carry the ChR2 (H134R)-EYFP gene in their Gt(ROSA)26Sor locus. The gene is separated from its CAG promoter by a loxP-flanked transcriptional STOP cassette, allowing its expression in a Cre-dependent manner (Madisen et al., 2012). All colonies were maintained onsite under controlled temperature (21–23°C) and photoperiod (12/12 h light/dark cycle 6 A.M. to 6 P.M.) while receiving ad libitum food (5L0D; LabDiet) and water access. Following OVX under 2% isoflurane, mice received a subcutaneous dose of 4–5 mg/kg carprofen (Rimadyl; Pfizer Animal Health) and then recovered for one week before experimentation.

### AAV delivery to POMC^Cre^ and POMC^Cre^::NPY^hrGFP^

Fourteen to 21 days (d) before each experiment, POMC^Cre^ or POMC^Cre^::NPY^hrGFP^ (>56 d old) received bilateral ARH injections of a Cre-dependent adeno-associated viral (AAV; serotype 1) vector encoding ChR2-mCh (AAV1-Ef1α-DIO-ChR2-mCherry, provided by Dr. Richard Palmiter, University of Washington, Seattle). Using aseptic technique, anesthetized (1-1.5% isoflurane/O_2_) mice received a medial skin incision to expose the surface of the skull. The glass pipette (#3-000-203-G/X; Drummond Scientific) with a beveled tip (diameter = 45 μm) was filled with mineral oil, loaded with an aliquot of AAV using a Nanoject II (Drummond Scientific). ARH injection coordinates were anteroposterior (AP): -1.18 mm, mediolateral (ML): ±0.33 mm, dorsoventral (DV): -5.80 and -5.70 (surface of the brain z = 0.0 mm); 250 nl of the AAV (2 × 10^12^ particles/ml) were injected (100 nl/min) at each position, the pipette was left in place for 10 min after injection and then slowly retracted from the brain. The skin incision was closed using skin adhesive, and each mouse received analgesia (Rimadyl; 4-5 mg/kg, s.c.).

### Estrous cyclicity

Mice were group housed with four to five mice/cage. Vaginal cytology was evaluated daily using a wet mount preparation. Briefly a small eye dropper was used to flush 0.1-ml saline into the vagina to recover surface cells. The sample was put on a clean glass slide and observed under the microscope. Some mice follow a 4- to 5-d cycle: proestrus (nucleated epithelial cells), estrus (cornified epithelial cells), and 2 d of diestrus (leukocytes). However, there is much less cycle regularity in mice as compared to other rodents and they can exhibit longer or shorter cycles (Cora et al., 2015). On observing a proestrous vaginal smear, we prepared the brain slices for single-cell harvesting and measured the uterine weight to confirm the estrous cycle stage. Only females with uterine weights >95 mg were included.

### Gonadectomy

Male gonads were left intact and all females were subjected to OVX at least 7 d before each experiment. Rimadyl (4–5 mg/kg, s.c.) was given immediately after surgery for relief of postoperative pain. Females received either an injection of sesame oil (50 μl, s.c.; Sigma-Aldrich) or a priming dose (0.125 μg/50 μl sesame oil, s.c.) of E2 benzoate (Sigma-Aldrich) on the Friday morning following surgery. In addition, oil or a low (0.25 μg) and then a high (1.5 μg) dose of E2 benzoate was administered in the morning of the 2 d preceding experiments. Circulating levels of E2 were verified by the uterine weights (<25 mg for OVX and >95 mg for E2 treated) at the time of hypothalamic slice preparation (between 8:30 and 10:30 A.M.).

### Single-cell harvesting and PCR (scRT-PCR)

Coronal brain sections (240 μm) were cut using a Vibratome (VT-1000; Leica) and the ARH was microdissected from basal hypothalamic slices (four slices per mouse) from POMC^eGFP^ (OVX females, *n* = 4; intact males mice, *n* = 2) and POMC^Cre::Ai32^ (OVX females, *n* = 3; intact males *n* = 4) for the single-cell experiments. For quantitative (q)RT-PCR experiments, intact, proestrous (*n* = 4, uterine weights 98–113 mg) or OVX female mice (*n* = 4), which were treated subcutaneously with oil (uterine weights: 19–24 mg) or E2 (uterine weights (102–111 mg), were used (*n* = 5 animals/group). Gentle trituration following incubation with protease (Sigma-Aldrich) was used to dissociate the ARH neurons. The dissociated neurons were dispersed onto a glass bottom dish and the healthy cells settled on and adhered to the glass bottom. After 15 min, the artificial CSF (aCSF) was removed, and fresh aCSF was added to the plate. This washing procedure was repeated two times. Throughout the dispersion and harvesting procedure, a constant flow (2 ml/min) of oxygenated aCSF circulated into the plate while the effluent circulated out using a peristaltic pump. The aCSF flow helped ensure fresh, oxygenated media was reaching the cells and assisted in clearing out unhealthy cells and debris from the trituration. The cells harvested were those observed to be fully intact, with one to three processes and a smooth cell membrane as visualized using an inverted microscope (DMIL; Leica) equipped with a fluorescent LED light source (X-Cite 110LED; Excelitas Technologies Corp.). Individual neurons that had adhered to the glass bottom dish were patched, and then harvested with gentle suction into the pipette using a XenoWorks Micromanipulator/Microinjector system (Sutter Instrument Company) and expelled into a siliconized 0.65-ml microcentrifuge tube containing Superscript III buffer (Invitrogen), 15-U RNasin (Promega), 10 mM dithiothreitol (DTT), and diethylpyrocarbonate (DEPC)-treated water in a total of 5 μl for scRT-PCR (one cell/tube) or 8 μl for qRT-PCR (10-cell pool/tube). After electrophysiological experiments, the cytosol of recorded cells was harvested with gentle suction into the recording pipette for *post hoc* identification with scRT-PCR. Each cell was expelled in a siliconized 0.65-ml microcentrifuge tube containing the solution described above. cDNA synthesis was performed in a reaction volume of 20 μl for single cells and 25 μl for cell pools containing dNTPs (0.5 mM, Promega), random primers (100 ng per tube, Promega), anchored oligo(dT)20 primers (400 ng/tube, Invitrogen), Superscript III reverse-transcriptase (100 U per tube, Invitrogen), RNAsin (15 U), DTT (6 mM), and DEPC-treated water according to the manufactures protocol (Superscript III, Invitrogen) and stored at -20°C. Controls included non-fluorescent cells, aCSF harvested in the vicinity of dispersed cells, water blank, single cells reacted without reverse transcriptase, and RNA extracted from hypothalamic tissue reacted with and without reverse transcriptase. Primers for the genes that encode NPY (*Npy*), AgRP (*Agrp)*, POMC (*Pomc*), vGluT2 (*Slc17a6*), vGAT (*Slc32a1*), μ-opioid receptor (*Oprm1),* VMAT2 (*Slc18A2*), and β-actin (*Actb*) were designed using Clone Manager software (Scientific & Educational Software). For sequences, please see Table 1.

**Table 1.** Primer Information

					Annealing	Efficiency		
Gene name (encodes for)	Accession number	Forward primer location (nt)	Reverse primer location (nt)	Product length (bp)	Temperature (°)	Slope	*r* ^2^	%
*Pomc* (POMC)^a^	NM_008895	145-164	327-344	200	60.5			
*Npy* (NPY)^a^	NM_023456	106-125	268-287	182	60			
*Agrp* (AgRP)^a^	NM_001271806	397-418	532-542	146	59			
*Oprm1* (muOR)^a^	NM_001302793	518-537	602-619	102	57			
*Slc17a6* (vGluT2)^a^	NM_080853	1038-1056	1213-1231	194	57			
*Slc17a6* (vGluT2)^b^	NM_080853	872-889	967-984	113	60	-3.293	0.92	100
*Slc32a1* (vGAT)^a^	NM_009508	813-834	928-949	137	60			
*Kiss1* (Kiss1)^a^	NM_178260	64-80	167-183	120	57			
*Slc18a2 (*VMAT2)^b^	NM_172523	1021-1041	1123-1143	123	60	-3.328	0.864	100
*Actb* (β-actin)^b^	NM_007393	446-465	535-555	110	60	-3.465	0.996	95

a primers used for scRT-PCR.

b primers used for qPCR.

All primers were designed to cross at least one intron-exon boundary. scPCR for individual cells from either acutely dispersed cells or cells collected after recording in the slice was performed on 3 μl of cDNA in a 20-μl reaction containing GoTaq buffer (5×, Promega), MgCl_2_ (2 mM, Promega), dNTPs (0.33 mM, Promega), forward and reverse primers (0.33 μM), Taq polymerase (2 U GoTaq, Promega), TaqStart antibody (Clontech). Fifty cycles of amplification were performed, and the PCR products were visualized with ethidium bromide on a 2% agarose gel.

qRT-PCR was performed on 4-μl duplicate samples for the target genes vGluT2 (*Slc17a6*), VMAT2 (*Slc18A2*), vGAT (*Slc32a1*), μ-opioid receptor (*Oprm1*), and on 2-μl duplicate samples for the reference gene β-actin (*Actb*) using Fast SYBR Green Master Mix (Applied Biosystems) in the Quant Studio 7 Flex Real-Time PCR System (Applied Biosystems).

### Visualized whole-cell patch recordings

Coronal brain slices (240 μm) containing the ARH from gonadectomized or intact mice were made in an ice-cold sucrose cutting solution (see recipe below) and stored in a bubbled chamber containing aCSF (see recipe below). Whole-cell patch recordings were performed in voltage clamp and current clamp using an Olympus BX51W1 upright microscope equipped with video-enhanced, infrared-differential interference contrast (IR-DIC) and an X-Cite 120 Series fluorescent light source (Excelitas Technologies Corp.). Electrodes were fabricated from borosilicate glass (1.5 mm OD; World Precision Instruments) and filled with a normal internal solution: 128 potassium gluconate, 10 NaCl, 1 MgCl_2_, 11 EGTA, 10 HEPES, 3 ATP, and 0.25 GTP (pH was adjusted to 7.3–7.4 with 1N KOH, 290–300 mOsm). High chloride internal solution consisted of 140 mM KCl, 5 mM MgCl_2_-6H_2_O, 1 mM MgCl_2_, 0.1 mM EGTA, 10 mM HEPES, 5 mM K_2_-ATP, and 0.35 mM Na_3_-GTP (pH was adjusted to 7.3–7.4 with KOH; 290–2945 mOsm). Cesium chloride internal solution consisted of 125 mM CsCl, 5 mM MgCl_2_, 1 mM BAPTA, 10 mM HEPES, 5 mM K_2_-ATP, and 0.4 mM Na-GTP (pH was adjusted to 7.3–7.4 with CsOH). Pipette resistances ranged from 3–5 MΩ. In whole-cell configuration, access resistance was <20 MΩ; access resistance was 80% compensated. For optogenetic stimulation, a light-induced response was evoked using an LED 470-nm blue light source controlled by a variable 2A driver (ThorLabs), with the light path delivered directly through an Olympus 40× water-immersion lens. High-fidelity response to light (470 nm) stimulation of POMC^ARH^ ChR2-expressing neurons was observed, and both evoked inward currents (in voltage clamp, V(hold) = −60 mV) or depolarization (in current clamp) were measured. Electrophysiological signals were amplified using the Axopatch 200B amplifier (Molecular Devices) and digitized using the Digidata 1440A digitizer (Molecular Devices), and the data were analyzed using p-Clamp software (RRID:SCR_011323, v10.3, Molecular Devices). The liquid junction potential was corrected for all data analysis.

### Solutions/drugs

A sucrose solution was used during Vibratome slicing: 2 mM KCl, 1 mM MgCl_2_-6H_2_O, 1.4 mM NaH_2_PO_4_, 10 mM HEPES, 10 mM glucose, 208 mM sucrose, 26 mM NaHCO_3_, 2 mM MgSO_4_-7H_2_O, and 1 mM CaCl_2_. Standard artificial cerebrospinal fluid was used: 124 mM NaCl, 5 mM KCl, 1.4 mM NaH_2_PO_4_, 5 mM HEPES, 10 mM glucose, 26 mM NaHCO_3_, 2 mM MgSO_4_-7H_2_O, and 2 mM CaCl_2_. All drugs were purchased from Tocris Bioscience unless otherwise specified. DAMGO (D-Ala2, N-MePhe4, Gly-ol]-enkephalin) was purchased from Peninsula Laboratories (Bachem).

### Immunocytochemistry (ICC) and imaging

POMC^Cre^ mice were injected bilaterally intra-ARH with AAV1-Ef1α-DIO-ChR2:mCherry as described above. Two to three weeks following the injection, mouse brains were prepared for ICC. Briefly, 2- to 3-mm coronal hypothalamic blocks were fixed by immersion in 4% paraformaldehyde, cryoprotected in 20% sucrose in Sorensen’s phosphate buffer, snap-frozen at -55°C, sectioned coronally on a cryostat at 20 μm, and thaw-mounted on Superfrost Plus slides (Thermo Fisher Scientific). Sections were rinsed in PB (0.1 M phosphate buffer, pH 7.4; all rinses were in PB for at least 30 min), and then incubated for 40 h at 4°C in a mixture of rabbit anti-β-endorphin primary antibody (1:2500; a generous gift from Dr. Robert Eskay; Dave et al., 1985; Rønnekleiv et al., 1990) and goat anti-mCherry primary antibody (1:10,000; Biorbyt). Subsequently, sections were rinsed in PB and incubated for 2–3 h at room temperature with a mixture of goat anti-rabbit Alexa Fluor 488 secondary antibody (1:500; Life Technologies) and bovine anti-goat Cy3 (1:300; Jackson ImmunoResearch). Finally, the sections were washed in PB and coverslipped using gelvatol containing the anti-fading agent, 1,4-diazabicyclo(2,2)octane (DABCO; Sigma-Aldrich; 50 mg/ml).

In select instances, at the end of the day following recordings Vibratome-sectioned slices were immersion fixed in 4% paraformaldehyde for at least 2 h, then rinsed in PBS, mounted, and coverslipped using gelvatol containing DABCO. All imaging was performed using a laser scanning confocal microscope (LSM 780; Zeiss) equipped with a 20× (numerical aperture 0.8) apochromatic objective and Zen software (Zeiss). For mCherry/Cy3, laser excitation was 561 nm and detection was 585–681 nm. GFP/Alexa Fluor 488 laser excitation was 488 nm and detection was 502–571 nm. Image processing was performed using FIJI (ImageJ) and Adobe Photoshop CC (Adobe Systems).

### Data analysis

#### Electrophysiology

ClampFit 10.3 (Molecular Devices) and Prism (GraphPad Software) were used for analysis. Comparisons between different treatments were performed using t-tests, where appropriate. Differences were considered statistically significant if *p* < 0.05. All data are expressed as mean ± SEM.

#### Real-time PCR

qPCR was performed on duplicate samples from six 10-cell pools from OVX (*n* = 4), proestrous (*n* = 4), oil-treated OVX (*n* = 5), and E2-treated OVX (*n* = 5) female mice. The relative linear quantity of the target gene was calculated using the formula 2^-ΔΔCT^ (Livak and Schmittgen, 2001). Data were expressed as an n-fold change in gene expression normalized to a reference gene (β-actin) and relative to the oil-control values and quantified using an unpaired Student’s *t* test.

#### Single-cell RT-PCR

**Figure 1.**
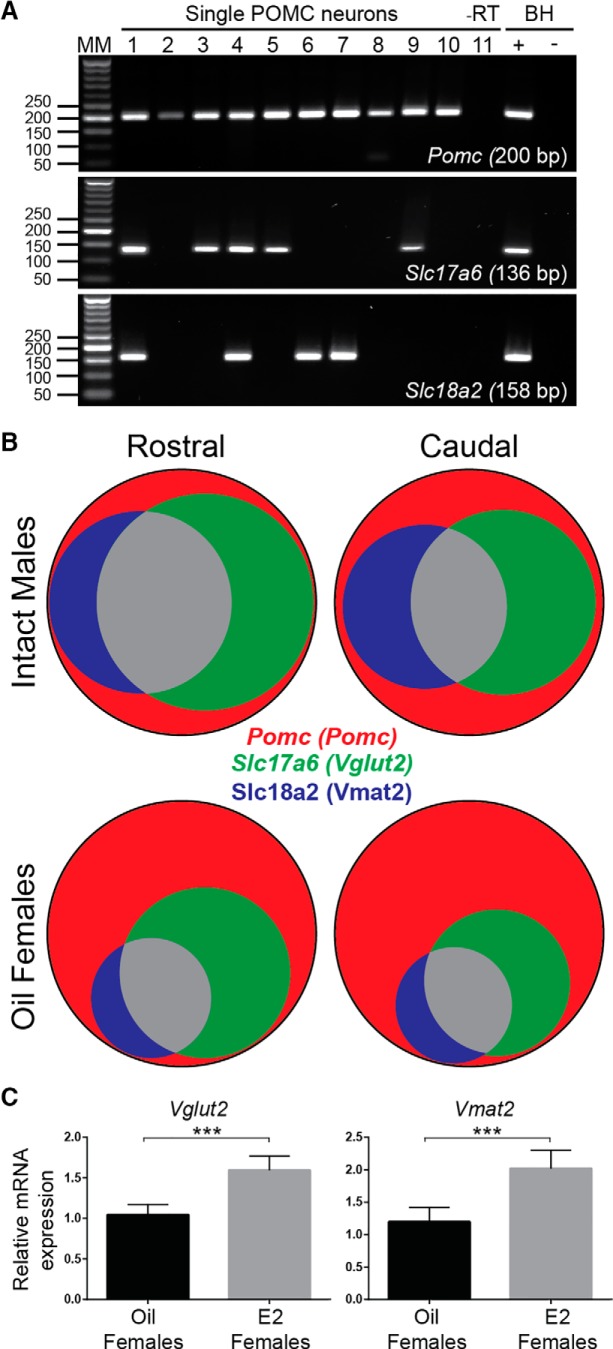
POMC neurons express mRNA for glutamate and GABA transporters. ***A***, Representative gels showing scRT-PCR detection of *Slc17a6 (Vglut2)* and *Slc18a2 (Vmat2)*, but not *Slc31a1 (Vgat)*, in individual POMC^eGFP^ neurons. ***B***, Venn diagrams of relative distribution of *Vglut2* and *Vmat2* mRNA in harvested POMC^eGFP^ cells from male mice (*n* = 2) and OVX females (*n* = 4). ***C***, qRT-PCR of 10-cell POMC^eGFP^ pools found that *Vglut2* and*Vmat2* mRNA was positively regulated by E2, as POMC^eGFP^ neurons from E2-treated females had significantly higher relative expression compared to OVX females (*t* test, *** *p* < 0.001, *n* = 5 animals/group).

For determination of POMC neuronal expression of a particular transcript, 20–40 cells/animal were harvested from OVX females and intact males. The number of cells expressing each transcript was counted for rostral and caudal ARH from each animal. The group mean ± SEM and percentage values were tabulated. Venn diagrams were generated using Venn Diagram Plotter (http://omics.pnl.gov/) to represent transcript coexpression (Fig. 1*B*).

**Table 2.** Statistics

	Test	*Post hoc*	Oil females	E2 females
Ai32 PPR	*t* test		14	11
Injected PPR	*t* test		12	13
STX	Paired *t* test		15	
Slow rescue	One-way ANOVA	Tukey’s		5
Naloxone	Paired *t* test			4
qRT-PCR	*t* test		4	4 (proestrus)
	*t* test		5	5

## Results

### POMC neurons express machinery for glutamate and GABA release

We began our examination of POMC neurotransmission using RT-PCR of single fluorescent cells from POMC^eGFP^ mice. This approach allowed for the sensitive and selective measurement of the transcripts supporting GABA and glutamate release. First, we chose to confirm that *Slc17a6,* the gene which codes for vesicular glutamate transporter 2 (vGluT2) was present. Next, we suspected that *Slc31a1* (*Vgat*) would not be found in POMC neurons, as previous studies were unable to detect vesicular GABA transporter (vGAT) mRNA or protein in POMC neurons (Ovesjö et al., 2001; Hentges et al., 2004). We hypothesized that an alternative transporter may be mediating non-canonical GABA release. Based on a report in dopamine neurons (Tritsch et al., 2012), *Slc18a2* (*Vmat2*), the transcript for the vesicular monoamine transporter, represented a strong candidate for this role.

Slices spanning the ARH were taken from POMC^eGFP^ OVX females (*n* = 4) and intact males (*n* = 2), dispersed and harvested as described in the Methods and the cells analyzed using scRT-PCR. As expected, *Vgat* was not detected, but in many *Pomc-*positive cells *Vmat2* and *Vglut2* mRNA were seen individually and coexpressed (Fig. 1*A*). For analysis, *Pomc* cells were segregated based on sex and relative location within the ARH (intact male rostral = 36 neurons and caudal = 27 neurons combined from the two males, OVX female rostral = 66 neurons and caudal = 71 neurons combined from the four females). These findings were not quantitative in nature but did provide a useful impression of the variability within POMC neurons. With this caveat in mind, POMC neurons from OVX females did appear to express less *Vglut2* and *Vmat2* mRNA than intact males (Fig. 1*A*,*B*), an indication that these transcripts were positively regulated by gonadal steroids. Therefore, to more accurately measure the effect of circulating estrogens on expression of these transporters in females, qPCR was performed on 10-cell pools (six 10-cell pools/mouse, *n* = 4 mice/group) of POMC^eGFP^ neurons from proestrous and OVX females. Between proestrous and OVX females we found no difference in the relative mRNA expression of V*mat2* (proestrous females: 0.94 ± 0.28 vs OVX females: 1.18 ± 0.35), but a 1.5-fold higher expression of *Vglut2* mRNA (proestrous vs OVX females: 1.59 ± 0.13 vs 1.07 ± 0.14, *p* < 0.05). Since OVX removes all ovarian hormones, including both E2 and progesterone, we repeated the experiment with pools from E2-treated OVX females instead of intact females (six 10-cell pools/mouse, *n* = 5 mice/group). Interestingly, both *Vglut2* (*p* < 0.001) and *Vmat2* (*p* < 0.001) relative mRNA expression was significantly lower in the oil-treated group (Fig. 1*C*). Therefore, *Vglut2* and *Vmat2* mRNA expression, like β-endorphin levels are increased with E2 treatment (Thornton et al., 1994; Bethea and Widmann, 1996). Furthermore, as the transporter copy number is associated with release probability of glutamate, and possibly GABA, through effects on the fill state (Herman et al., 2014), one could anticipate greater POMC synaptic efficacy in E2-treated OVX females compared to oil-treated OVX counterparts.

### POMC input to NPY neurons

**Figure 2.**
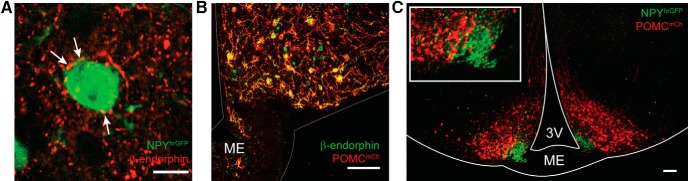
Virally mediated expression of channelrhodopsin in POMC^Cre^ mice is restricted primarily to POMC neurons. ***A***, Confocal image of immunocytochemical staining using an antibody directed against β-endorphin showed close contact with NPY^hrGFP^ neurons in the ARH. Scale bar: 10 μm. ***B***, Confocal image (20×) of a 20-μm cryostat section of POMC^Cre^::ChR2:mCherry that underwent ICC with antibodies against β-endorphin (green) and mCherry (red). High degree of colocalization indicates that virally driven ChR2-mCherry expression is specific to POMC neurons. Scale bar: 100 μm. ***C***, Confocal image of a 240-μm coronal brain slice from a NPY^hRGFP^ (green) × POMC^Cre^::ChR2:mCherry (red) mouse. These arcuate populations are distinct both geographically and morphologically. Scale bar: 100 μm.

While an early examination using channelrhodopsin (ChR2) assisted circuit mapping suggests that POMC neurons do not directly project to NPY/AgRP neurons (Atasoy et al., 2012), preliminary confocal analysis of ICC labeling of slices taken from a NPY^hrGFP^ mouse found β-endorphin fibers made close contact with NPY neurons (Fig. 2*A*). Rather than a general survey of ARH interactions or POMC release, we sought to focus on the POMC to NPY synapse. Therefore, we used a POMC^Cre^ mouse line in conjunction with an AAV1-Ef1α-DIO-ChR2-mCherry virus. Due to concerns over specificity (Padilla et al., 2010), we began with immunocytochemical and histologic examinations to validate the model. AAV injections were made bilaterally into the ARH of adult POMC^Cre^ mice. Animals were killed two weeks later, a block containing the ARH was fixed, and 20-μm sections cut on a cryostat. ICC was then performed on slides with antibodies for β-endorphin and mCherry (mCh). Confocal imaging showed that mCh expression was only seen in cells labeled by the β-endorphin antibody (Fig. 2*B*). Next, additional AAV-injected brains (*n* = 2) were extracted, Vibratome-sectioned at 240 μm, and immersion fixed before being mounted on slides and coverslipped. NPY^hrGFP^ and POMC^Cre^::ChR2-mCh cells were seen as distinct populations, differing in location within the ARH and typical soma size (Fig. 2*C*). Therefore, AAV-mediated channelrhodopsin expression is specific in adult POMC^Cre^ mice.

**Figure 3.**
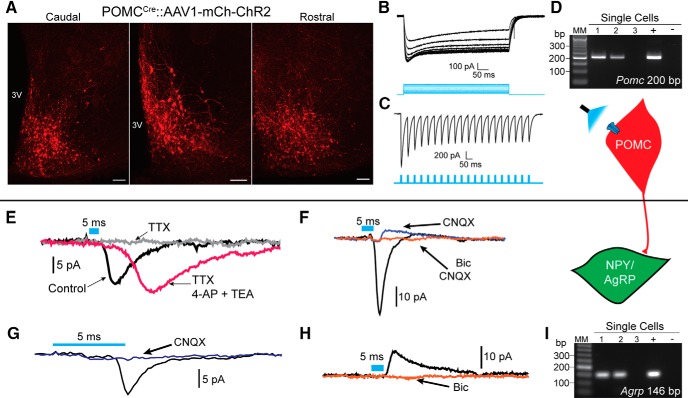
Optogenetic activation of POMC neurons produces postsynaptic responses in NPY/AgRP neurons. ***A***, Confocal images (10×) of serial 240-μm coronal brain slices from an ARH-injected POMC^Cre^::ChR2:mCherry mouse. ***B***, Optogenetic (470-nm light) stimulation produced strong, sustained inward currents. ***C***, Channelrhodopsin currents in POMC neurons are able to faithfully follow 20-Hz optogenetic stimulation. ***D***, Single cells harvested from fluorescent cells that responded with direct channelrhodopsin currents. ***E***, Postsynaptic nature of responses was confirmed when they were rescued from 1 μM TTX after coapplication of 0.5 mM 4-AP and 7.5 mM TEA. ***F***, Both CNQX-sensitive, inward, and bicuculline-sensitive, outward currents were detected. ***G***, Using a standard K^+^ gluconate internal solution and holding V_m_ at -60 mV, glutamatergic currents were primarily encountered. ***H***, Less frequently, GABAergic currents could be observed. Traces are the average of 50 sweeps, blue bars representing optogenetic stimulation are 5 ms for postsynaptic responses. Scale bars: 100 μm. ***I***, Single cells harvested following recordings which displayed a postsynaptic current. The majority could be identified as NPY/AgRP neurons *post hoc* for inclusion in subsequent analyses.

Once again, POMC^Cre^ mice were given a bilateral ARH injection of AAV1-Ef1α-DIO-ChR2-mCherry to drive expression of ChR2:mCh; Fig. 3*A*). During the week following injection, female mice underwent OVX and subsequently *sc* injected with either oil or E2 while males were left intact. Electrophysiology experiments were conducted 14–28 d post-viral injection. Fluorescent POMC^mCh^ neurons responded to 5 ms of 470 nm light with inward currents, which persisted throughout the stimulus duration (100–500 pA; Fig. 3*B*), and were able to easily follow 20 Hz stimulation (Fig. 3*C*), similar to what has been recently described for kisspeptin (Kiss1) neurons (Qiu et al., 2016). Cells with these direct ChR2 responses had an average input resistance of 980 ± 150 MΩ (*n* = 15). A lower average input resistance compared to NPY/AgRP neurons was consistent with previous findings (Smith et al., 2013; Qiu et al., 2014). The cytosol of several neurons exhibiting direct ChR2 currents were harvested and most were found to express *Pomc*, and none *Agrp/Npy* using scRT-PCR (Fig. 3*D*).

To assess the ability of POMC neurons to release GABA and glutamate onto NPY/AgRP neurons, we performed whole-cell voltage clamp recordings from NPY/AgRP neurons in intact males, oil-treated OVX females, and E2-treated OVX females using optogenetic stimulation. NPY/AgRP neurons were targeted based on either their fluorescence (hrGFP) or small soma size and proximity to the median eminence. We have found that medially located NPY/AgRP neurons can be readily segregated from the more lateral POMC neurons based on their high input resistance and low capacitance (Smith et al., 2013). In addition, when using POMC^Cre^ mice, the cytosol from recorded neurons was harvested and used for *post hoc* RT-PCR confirmation of *Agrp* (or *Npy)* expression (Fig. 3*I*). Cells that did not test positive for *Npy/Agrp* and/or had an input resistance <1 GΩ and capacitance >18 pF were excluded from analysis. To confirm the postsynaptic nature of these responses, currents were blocked by 1 μM tetrodotoxin (TTX) and rescued with 0.5 mM 4-AP and 7.5 M TEA through facilitation of channelrhodopsin-mediated depolarization of nerve terminal and transmitter release (Fig. 3*E*; Cousin and Robinson, 2000; Petreanu et al., 2009). At V_m_ = -60 mV, two of the recordings exhibited mixed responses following low-frequency stimulation, both inward and outward currents in a single neuron (Fig. 3*F*), with the inward current always preceding the outward currents. Mixed responses could have arisen from multi-synaptic input or corelease, which using full field illumination we could not differentiate. The corelease would be consistent with previous studies, which show that optogenetic stimulation of POMC^Cre^::ChR2 neurons releases both GABA and glutamate (Atasoy et al., 2008; Dicken et al., 2012), as well as with the presence of both *Vglut2* and *Vmat2* transcripts in a subset of POMC neurons. However, the majority (88.4%) of currents were inward currents and blocked by CNQX (Fig. 3*G*), indicating that they were glutamatergic, AMPA receptor mediated. Only 7.5% of the observed postsynaptic responses were outward and were eliminated with bath application of bicuculline (Fig. 3*H*). Outward currents were observed using a standard internal solution and holding the cell at -60 mV. At times, antagonism of the inward current with CNQX and/or holding V_m_ at -10 mV revealed a more prominent GABA response. This suggests that GABAergic currents may be masked by glutamatergic responses in the present study. If true, the tail, but not the peak, of inward currents may be reduced by the slower inhibitory input. To address this possibility, we used a high chloride internal solution in conjunction with constant bath perfusion of 50 μM D-AP5 and 10 μM CNQX. Despite this procedure, we did not see an increase in the rate at which GABAergic currents were encountered (only one out of 34 ARH cells). However, the high chloride greatly enhanced the frequency of spontaneous events, making detection of evoked responses difficult. Similarly, a cesium chloride internal solution caused large, non-evoked events without an appreciable improvement in the ability to detect GABAergic currents. Ultimately, however, we sought to investigate the ability of E2 to modulate neurotransmission from POMC cells and only the fast kinetics of AMPA receptors, not GABA_A_, are suitable for assessing changes in release probability by measuring the paired pulse ratio (PPR; Kaeser and Regehr, 2014; Nestor et al., 2016).

### E2 increases the probability of glutamate release

**Figure 4.**
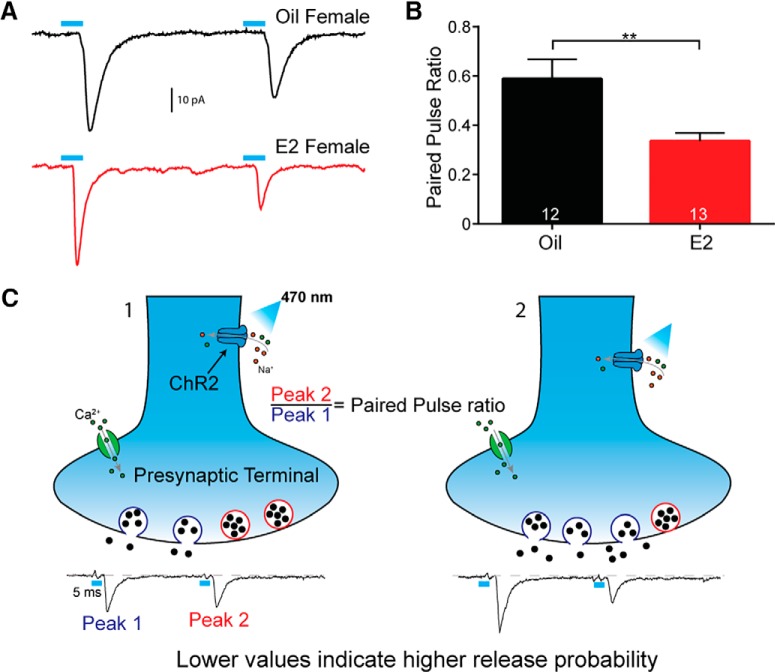
Estradiol-treated OVX females have an increased probability of glutamate release onto NPY/AgRP neurons. ***A***, Representative traces from E2-treated and oil-treated OVX female POMC^Cre^::ChR2:mCherry mice. Paired pulse protocol uses 5-ms 470-nm optogenetic stimulation with an interstimulus interval of 50 ms to measure release probability. The PPR is equal to the amplitude of peak 2 over peak 1. Lower numbers indicate a higher release probability. ***B***, PPR is significantly lower with E2 treatment compared to oil-treated OVX mice. Traces are the average of 50 sweeps (*t* test, ** *p* < 0.01). ***C***, Schematic illustrating how the PPR provides a measure of neurotransmitter release probability. In terminal 1, when the two peaks are of a similar amplitude, the first stimulus did not deplete the readily releasable pool of vesicles. In terminal 2, the first stimulus caused nearly all of the vesicles to fuse and release neurotransmitter, leaving little for the next stimulus.

Based on the increased *Vglut2* mRNA expression in E2-treated OVX and intact proestrous females, we hypothesized that the anorexigenic effects of E2 were mediated, in part, through enhancement of POMC neurotransmission. Therefore, once again we administered a series of subcutaneous E2 injections to mimic the proestrous surge (Bosch et al., 2013). To measure changes in synaptic efficacy we employed a paired pulse paradigm, in which brief stimuli (5 ms, 470 nm light pulses) were quickly delivered with a short (50 ms) interstimulus interval (Jackman et al., 2014; Fig. 4*A*). The amplitude of the second peak was divided by the amplitude of the first peak to calculate the PPR. This provided a normalization that accounted for variation in expression of ChR2, allowing for both between and within subject comparisons. Lower numbers are associated with a high probability of neurotransmitter release, whereas a PPR near one represents low synaptic efficacy (Herman et al., 2014; Nestor et al., 2016). Indeed, we found that E2 treatment led to a significant decrease in the PPR [E2-treated (*n* = 13): 0.34 ± 0.03 vs oil-treated (*n* = 12): 0.59 ± 0.08, *t* test, *p* < 0.01; Fig. 4*B*]. Therefore, E2 appears to increase the efficacy of POMC to NPY/AgRP transmission (Fig. 4*C*).

### Acute activation of mERs rapidly increases probability of glutamate release

Here, we used POMC^Cre^::Ai32 mice, as they did not require viral injections. A concern was that Cre-dependent expression of the ChR2::eGFP fusion protein could be driven in non-POMC neurons embryonically, with Cre and/or ChR2::eGFP expression persisting into adulthood (Padilla et al., 2010). Based on harvesting 270 individual fluorescent cells (intact males, *n* = 4 and OVX females, *n* = 3) from POMC^Cre^::Ai32 ARH brain slices, we found 70% expressed *POMC* while 14% expressed *Agrp* and the remaining 16% expressed neither transcript. Therefore, the majority of neurons containing the ChR2::eGFP protein were POMC. Furthermore, by focusing on glutamatergic input we greatly reduced the possibility of studying release from non-POMC neurons as NPY/AgRP do not release glutamate (Atasoy et al., 2012). Also, PPR data from POMC^Cre^::Ai32 mice was congruent with AAV-injected POMC^Cre^ mice as oil-treated OVX females exhibited the largest PPR (0.70 ± 0.07, *n* = 14), and the E2-treated OVX females showed the lowest PPR (0.28 ± 0.04, *n* = 11).

**Figure 5.**
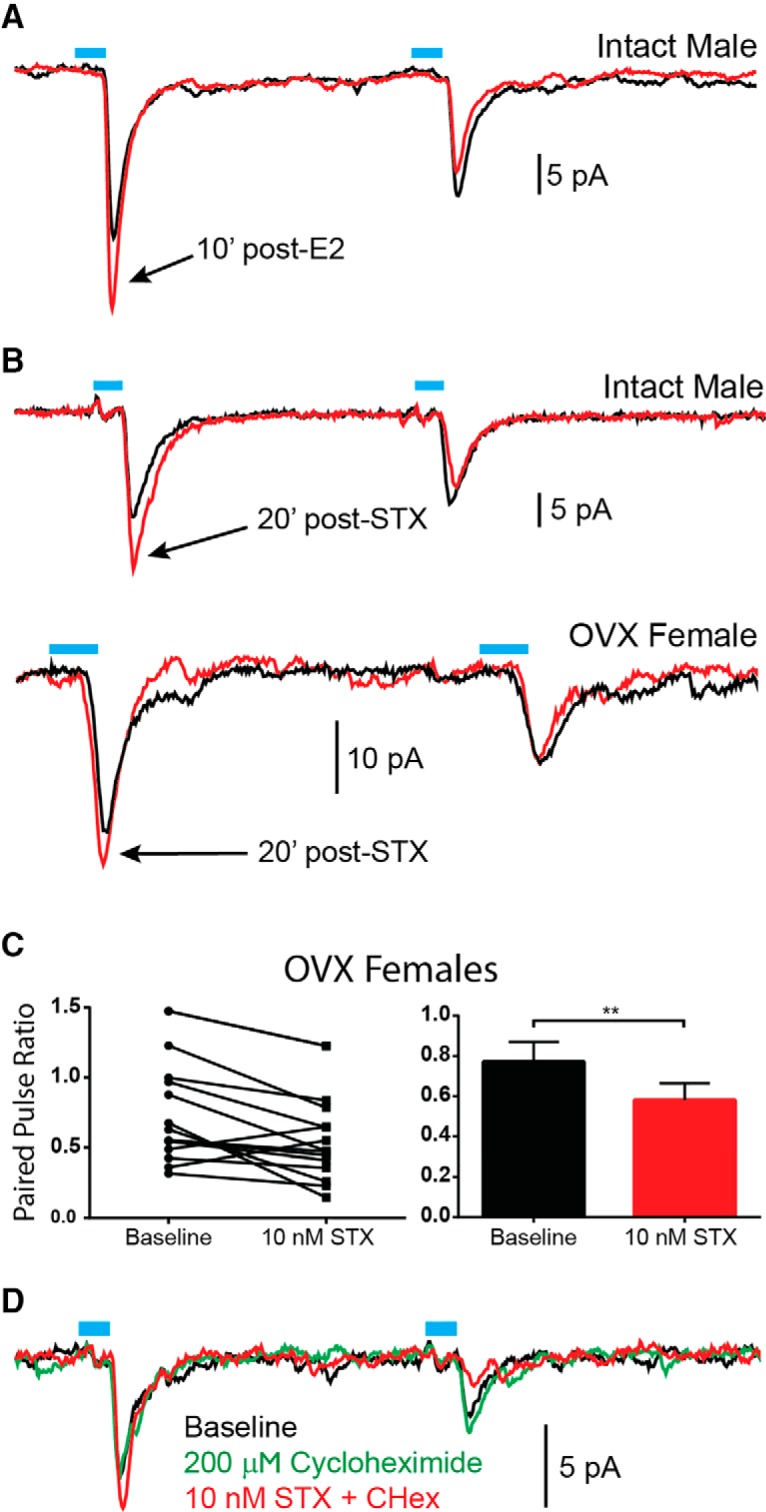
Acute activation of estrogen receptors (ERs) increases release probability from POMC neurons through a rapid, non-genomic mechanism. ***A***, Within 10 min of E2 (100 nM) addition to the bath, the PPR is decreased, that is release probability is increased. The rapidity of this effect suggests a non-genomic mechanism of action. ***B***, Bath application of STX (10 nM), an agonist selective for a Gq-coupled membrane ER, was similarly able to decrease the PPR in cells from intact males and OVX females. ***C***, The decrease in PPR following addition of STX to the bath was statistically significant in OVX females (paired *t* test, ** *p* < 0.01). ***D***, Pre-treatment with cycloheximide did not prevent the effects of STX, indicating changes in transcription/translation were not necessary. Traces are the average of 50 sweeps.

To test the effect of acute estrogen receptor (ER) activation, we perfused 100 nM E2 to the bath and found that the PPR was decreased within 10 min in slices taken from intact males (*n* = 2; Fig. 5*A*). Further assessing the involvement of membrane-initiated signaling of estrogen (Hammes and Levin, 2007), we used STX, a selective ER agonist that acts via a putative membrane G_q_-coupled ER (G_q_-mER; Qiu et al., 2003, 2006). STX desensitizes presynaptic POMC GABA_B_ receptors, which is known to increase release probability through disinhibition of voltage-gated Ca^2+^ channels, and POMC neurons seem to universally respond to STX (Qiu et al., 2003, 2006; Conde et al., 2016). We used the longer application time compared to E2 to compensate for the slower pharmacokinetics due to the 10-fold lower concentration of STX. The higher concentration of E2 was selected to ensure rapid penetration into the slice, whereas a lower concentration was used for the more potent STX (Qiu et al., 2006). Indeed, 10 nM STX appeared efficacious, in both intact males and OVX females, in decreasing the PPR (Fig. 5*B*).

To confirm this effect, slices were taken from oil-treated OVX females to maximize potential acute effects of STX. E2-treated females were not tested due to the already low PPR, which would likely cause a floor effect, as E2 would bind and occlude the G_q_-mER. After 20 min of STX bath perfusion, 12 cells showed a decreased PPR that was statistically significant using a paired *t* test (*n* = 15, *p* < 0.001; Fig. 5*C*). To be certain that gene transcription was not involved and a rapid, non-genomic mechanism mediated this effect, cells were pre-treated with cycloheximide for 15 min before application of STX to the bath (Lagrange et al., 1997). Cycloheximide prevents protein synthesis, therefore, any effects observed must be non-genomic. Indeed, STX (*n* = 2) was still able to decrease the PPR demonstrating that activation of a Gq-coupled mER is sufficient to increase release probability from POMC onto NPY/AgRP neurons (Fig. 5*D*).

### High-frequency stimulation releases beta-endorphin

**Figure 6.**
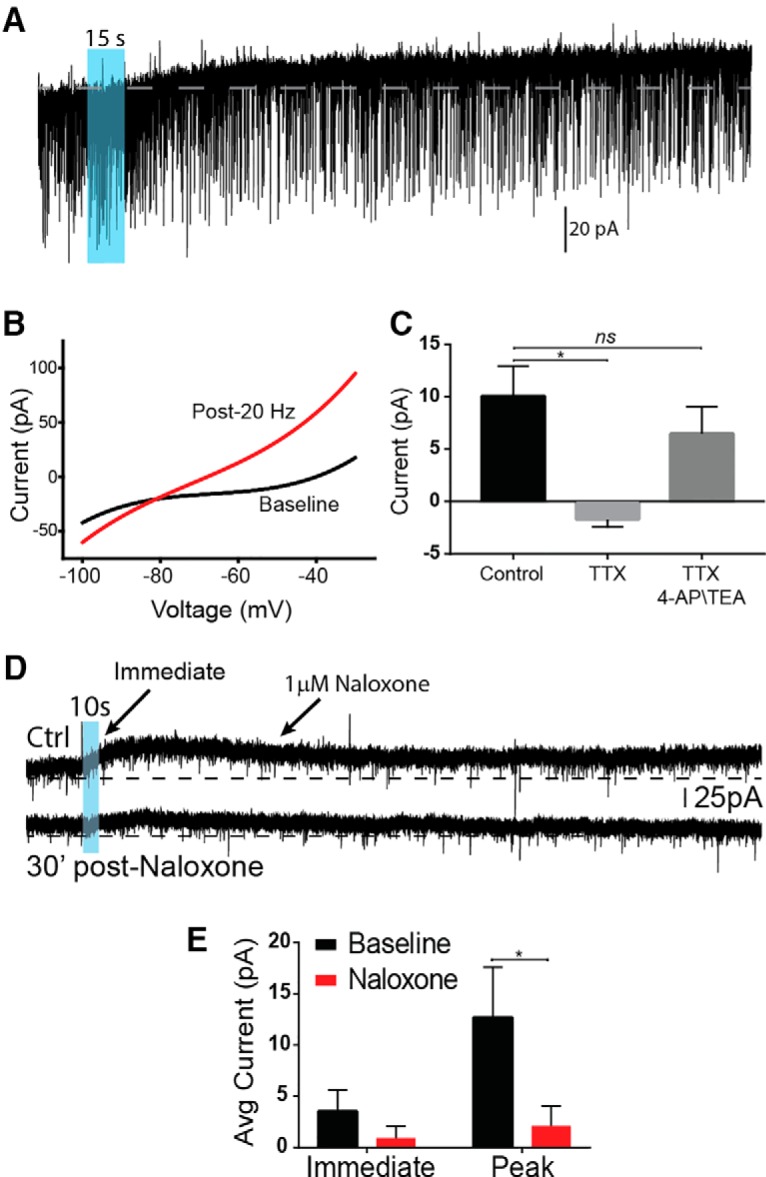
High-frequency stimulation of POMC neurons causes release of β-endorphin. ***A***, 20 Hz (10 ms pulse width) for 15 s of 470 nm light could elicit a slow, long-lasting outward current. ***B***, Following high-frequency stimulation, the I/V curve crossed closer to E_K_^+^ (average -82.7 ± 1.6 mV) and exhibited inward rectification, suggesting GIRK channels had been activated. ***C***, High-frequency response could be blocked with TTX and recovered with addition of 0.5 mM 4-AP and 7.5 mM TEA (one-way ANOVA, *p* < 0.05, Tukey’s *post hoc*). ***D***, ***E***, The response to high-frequency stimulation is antagonized by bath application of 1 μM naloxone, suggesting that β-endorphin is acting on postsynaptic opioid receptors. ***D***, Bar graphs showing naloxone block on the immediate and peak response following high-frequency stimulation (*n* = 4, average over 10-s period). Paired *t* test found the peak amplitude was significantly inhibited by naloxone (paired *t* test, * *p* < 0.05).

Previous reports indicate that high-frequency optogenetic stimulation can elicit peptide release (Piñol et al., 2012; Kempadoo et al., 2013; Walsh et al., 2014; Apergis-Schoute et al., 2015; Qiu et al., 2016), and ARH neurons fire at 20-Hz frequencies *in vivo* (Moss et al., 1975). Therefore, we implemented a 20 Hz, high-frequency stimulus protocol to determine if optogenetics can be used to drive POMC neuropeptide transmission. As POMC neurons produce both α-melanocyte-stimulating hormone (α-MSH; excitatory, via a G_s_-coupled receptor) and β-endorphin (inhibitory, via a Gα_i/o_-coupled receptor; Clark et al., 2006; Silva et al., 2001), both inward and outward currents were possible. However, optogenetic stimulation of POMC^Cre^::AAV-ChR2-mCh neurons predominantly elicited outward currents (Fig. 6*A*) with immediate responses of ∼3 pA over the 10 s immediately following stimulation (Fig. 6*A*). Maximum outward current was typically reached 1–2 min following stimulation in NPY/AgRP neurons (10.3 ± 1.3 pA, *n* = 23), but was monitored for at least 5 min. Interestingly, the I/V for the response reversed at ∼E_K_^+^ (-82.7 ± 1.6 mV, *n* = 7) and exhibited inward rectification, indicative of μ-opioid receptor activation of GIRK channels (Fig. 6*B*). In isolation, these findings do not unequivocally demonstrate direct action of release from POMC onto NPY/AgRP neurons. Therefore, we first elicited a slow response to high-frequency optogenetic stimulation. Next, 1 μM TTX was added to the bath to block voltage activated Na^+^ channels, isolating the cell. Finally, 0.5 mM 4-AP and 7.5 mM TEA were applied in the continued presence of TTX to block K^+^ channels and facilitate ChR2-mediated depolarization of terminals and neurotransmitter release while preventing a multisynaptic response to high-frequency stimulation (Qiu et al., 2016). After 25 min, high-frequency stimulation was once again able to cause a slow outward current (*n* = 5; Fig. 6*C*), which is further evidence for a direct input to NPY/AgRP neurons (ANOVA, *p* < 0.05).

**Figure 7.**
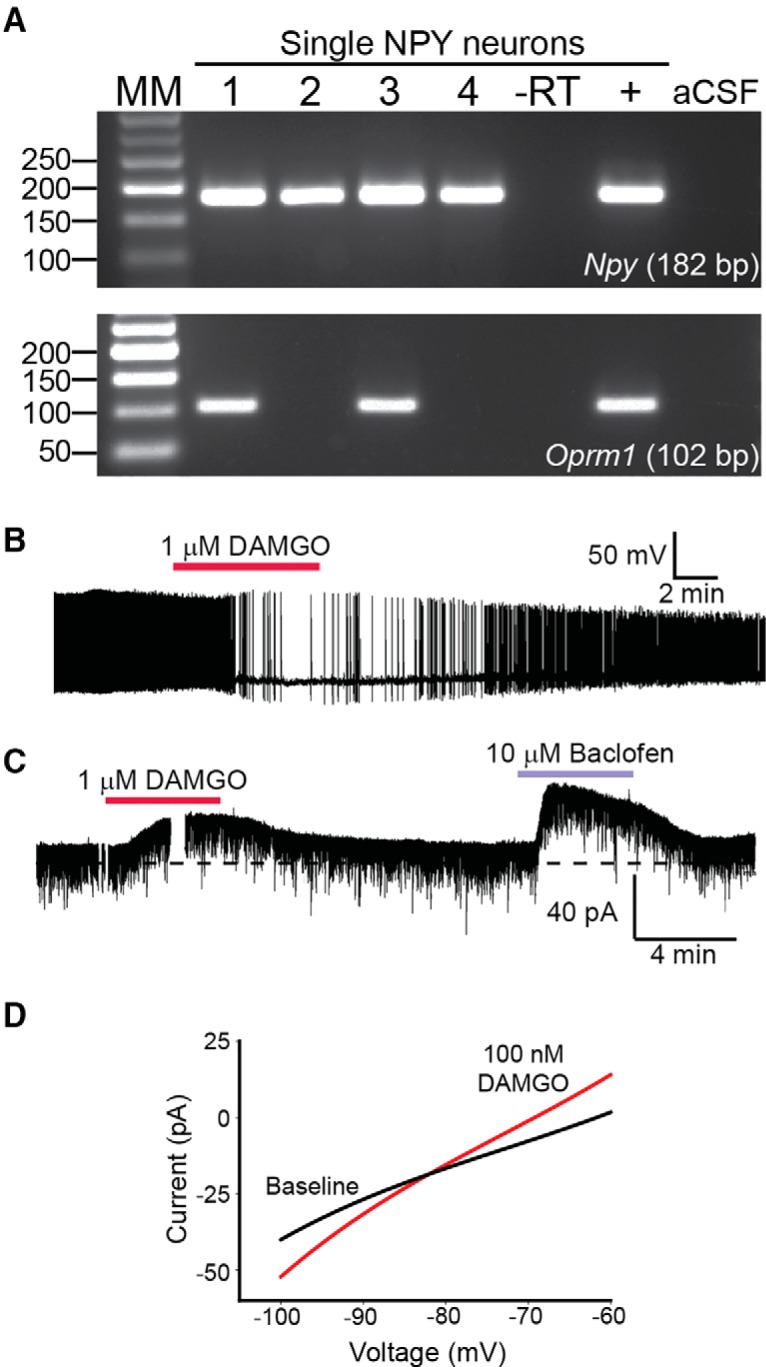
μ-Opioid signaling in single NPY neurons. ***A***, Representative gel showing that *Oprm1* the transcript for the μ-opioid receptor could be detected in ∼40% of single NPY^hrGFP^ neurons. ***B***, Bath application of 1 μM DAMGO greatly diminished the firing of action potential in an NPY neuron under current clamp. ***C***, Both 1 μM DAMGO and 10 μM baclofen produced strong outward currents in voltage clamp when added to the bath. ***D***, Following bath application of DAMGO, the outward current reversed near -90 mV.

To better establish the nature of the postsynaptic response, we tested to see whether naloxone, an opioid antagonist, could block or attenuate the outward current. Indeed, addition of 1 μM naloxone (*n* = 4) to the bath for 20 min antagonized subsequent attempts to evoke a slow response and significantly decreased the peak outward current (*p* < 0.05), indicating that β-endorphin released from POMC neurons inhibited postsynaptic NPY/AgRP neurons via activation of opioid receptors (Fig. 6*D*,*E*). In addition, we tested for the expression of *Oprm1*, the transcript for μ-opioid receptor, in single NPY^hrGFP^ neurons. *Oprm1* mRNA was detected in NPY^hrGFP^ cells from E2-treated OVX females (40.0 ± 4.6%, 25 cells/mouse, *n* = 3; Fig. 7*A*). Therefore, at least a subset of NPY/AgRP neurons express μ-opioid receptors that are activated following high-frequency stimulation of POMC neurons.

Next, we used DAMGO, a μ-opioid selective agonist, to measure the effect of activation of this receptor in NPY^hrGFP^ neurons. In current clamp, 1 μM DAMGO inhibited action potential firing by 90% (Fig. 7*B*). For comparison to high-frequency outward EPSCs, we made voltage clamp recordings in the presence of 1 μM TTX while perfusing 1 μM DAMGO or 10 μM baclofen, observing outward currents of ∼10 and 40 pA, respectively (Fig. 7*C*), both of which exhibited reversal potentials near EK^+^ (Fig. 7*D*). While we began with 1 μM, to establish an effect, μ–opioid receptors display acute desensitization (Harris and Williams, 1991) and, therefore, we proceeded with lower concentrations for our examination of dose response. We made voltage clamp recordings from NPY^hrGFP^ neurons in the presence of 1 μM TTX after addition of 100, 300, and 600 (*n* = 11) nM DAMGO, observing outward currents of 13.3 ± 4.3, 22.7 ± 4.1, and 28.8 ± 4.5 pA, respectively. Following administration of 600 nM DAMGO, 10 μM baclofen was bath applied as a positive control, and any cells which did not respond were excluded from the analysis. Together, these findings strongly suggest that highly active POMC neurons, as in high E2-states (Kelly et al., 1992), will release β-endorphin onto NPY/AgRP neurons, inhibiting them through activation of μ-opioid receptors. As mentioned, a small number of the high-frequency responses were slow inward currents (i.e., excitatory) presumably mediated through melanocortin signaling. This minority of responses (*n* = 3/26) had a peak amplitude of -8.6 ± 2.6 pA. However, *post hoc* identification by scRT-PCR found only one cell to be NPY/AgRP, with the others being Kiss1 cells. Therefore, β-endorphin is the primary peptide (95.8%) released onto NPY/AgRP neurons during high-frequency optogenetic stimulation of POMC neurons. This suggests that POMC neuropeptides may be released in a segregated manner (Vaaga et al., 2014).

## Discussion

A long-standing hypothesis has been that the anorexigenic activity of E2 is mediated largely through POMC neurons, which have been considered integral to the central control of energy homeostasis (Qiu et al., 2003, 2006; Xu et al., 2011). Herein, we determined that POMC neurons express *Slc17a6* (*Vglut2*) and *Slc18a2* (*Vmat2*), and qRT-PCR of pooled POMC neurons revealed that *Vglut2* and *Vmat2* mRNA expression was augmented by E2 in OVX mice. E2 also increased the probability of glutamate release onto NPY/AgRP neurons following optogenetic stimulation of POMC neurons. These effects of E2 were recapitulated by bath application of the G_q_-mER selective ligand STX, indicative that these pre-synaptic effects of E2 were mediated by a membrane ER. Moreover, high-frequency stimulation of POMC neurons released β-endorphin that inhibited NPY/AgRP neurons through activation of μ-opioid receptors coupled to GIRK channels. This projection and the reciprocal connection with NPY/AgRP neurons will amplify changes in activity of each (NPY-POMC) population, as excitation of one will lead to inhibition of the other. In particular, this circuit would serve the proposed “Flip-Flop” model wherein a set point is defended to prevent unnecessary adjustments to behavior due to minor fluctuations in energy balance (Yang et al., 2011). Therefore, E2 drives the anorexigenic activity of POMC neurons by increasing their excitability (Roepke et al., 2011b; Smith et al., 2014) and facilitating inhibitory synaptic input to NPY/AgRP neurons.

We began our investigation by assessing the presence of transcripts necessary for GABA and glutamate release in individual POMC^eGFP^ cells. In agreement with earlier studies, we confirmed that POMC neurons did not express *Vgat* mRNA (Ovesjö et al., 2001). Yet, a subset of POMC^eGFP^ cells in culture has been shown to release GABA and express *Gad* mRNA (Hentges et al., 2004). The Hentges group also documented that GAD67^GFP^ is present in nearly half of fluorescent POMC neurons, particularly in the caudal ARH (Hentges et al., 2009) and that optogenetic stimulation of POMC neurons tended to produce GABAergic, more often than glutamatergic, responses in unidentified cells (Dicken et al., 2012). Therefore, we suspected the involvement of a vGAT-independent mechanism of GABA release and selected VMAT2 as a likely candidate based on its support of non-canonical GABA release in dopamine neurons (Tritsch et al., 2012). Similar to POMC expression of *Gad* mRNA (Hentges et al., 2004), we found approximately a third of POMC^eGFP^ neurons expressed *Vmat2* mRNA. POMC neurons therefore possess the necessary enzymes and transporters to release GABA. However, despite the presence of *Vmat2* and previous reports of POMC GABA release, we were surprised to observe only infrequent IPSCs in NPY/AgRP neurons, as a GABAergic input from POMC neurons would provide a straightforward means to reduce food intake. Therefore, an effort was made to enhance and isolate IPSCs with perfusion of CNQX and D-AP5 in conjunction with different internal solutions, regardless GABAergic IPSCs remained rare. Interestingly, IPSCs were more frequent in unidentified neurons within 100 μm of ChR2 expressing POMC neurons in a sagittal slice preparation (Dicken et al., 2012). Such an approach might have reduced the chances of patching NPY/AgRP neurons, but together with our findings would suggest that while POMC neurons do release GABA these projections are sent elsewhere and not onto NPY/AgRP neurons.

With regards to glutamatergic signaling, we found that about half of all POMC neurons expressed *Vglut2* mRNA, and the majority of PSCs we recorded in NPY/AgRP neurons were glutamatergic. On the surface this input may appear counterproductive to the anorexigenic function of POMC neurons. However, *Vglut2* mRNA expression varied with the stage of the estrous cycle and was increased with E2-treatment. Congruent with the qPCR data, E2-treated OVX females had a lower average PPR (highest synaptic efficacy) compared to oil-treated OVX females. We propose that during periods of high circulating E2, which increases the activity of POMC neurons (Kelly et al., 1992), greater release of glutamate from POMC neurons will exert an inhibitory influence on NPY/AgRP neurons in part through activation of G_i/o_-coupled Group II/III metabotropic glutamate receptors (mGluRs), which are known to be expressed in NPY/AgRP neurons and activated by high-frequency stimulation (Nestor et al., 2016; Qiu et al., 2018). Alternatively, the reduced glutamate release could primarily activate AMPA and NMDA receptors in NPY/AgRP neurons to stimulate food intake when the concentration of circulating estrogens are low and/or the animal is in a fasted state (Liu et al., 2012).

High-frequency optogenetic stimulation can be leveraged to study peptide release (Piñol et al., 2012; Kempadoo et al., 2013; Walsh et al., 2014; Apergis-Schoute et al., 2015; Qiu et al., 2016). Here we report for the first time that POMC neurons transmit peptidergic (β-endorphin) inhibitory input to NPY/AgRP neurons. High-frequency (20 Hz) optogenetic stimulation for 10–15 s elicited a slow outward current and produced an I/V that exhibited the tell-tale characteristics of GIRK channel activation (Smith et al., 2013). While the sensitivity to naloxone suggests that the majority of this current was mediated via opioid receptors, the residual component could represent a contribution by Group II/III mGluRs since many of the cells exhibiting a high-frequency response also displayed EPSCs to low-frequency stimulation. Given that *in vivo* ARH POMC neurons can fire at such high frequencies (Moss et al., 1975), this likely represents physiologically relevant transmission. In addition, based on scRT-PCR analysis, we determined that NPY/AgRP neurons express *Oprm1*, the transcript for the μ-opioid receptor, and DAMGO, a μ-opioid selective agonist, produced a strong outward current. Together, these results indicate that the outward current in NPY/AgRP neurons following high-frequency optogenetic stimulation of POMC neurons was generated, at least in part, by β-endorphin binding to μ-opioid receptors, which activated GIRK channels (Loose and Kelly, 1990). In this respect, perhaps E2 protects females from weight gain by increasing β-endorphin signaling (Thornton et al., 1994; Bethea and Widmann, 1996). This could explain why selective deletion of *Vglut2* from POMC neurons only affected weight gain in males, not females, following exposure to a high-fat diet (Dennison et al., 2016).

**Figure 8.**
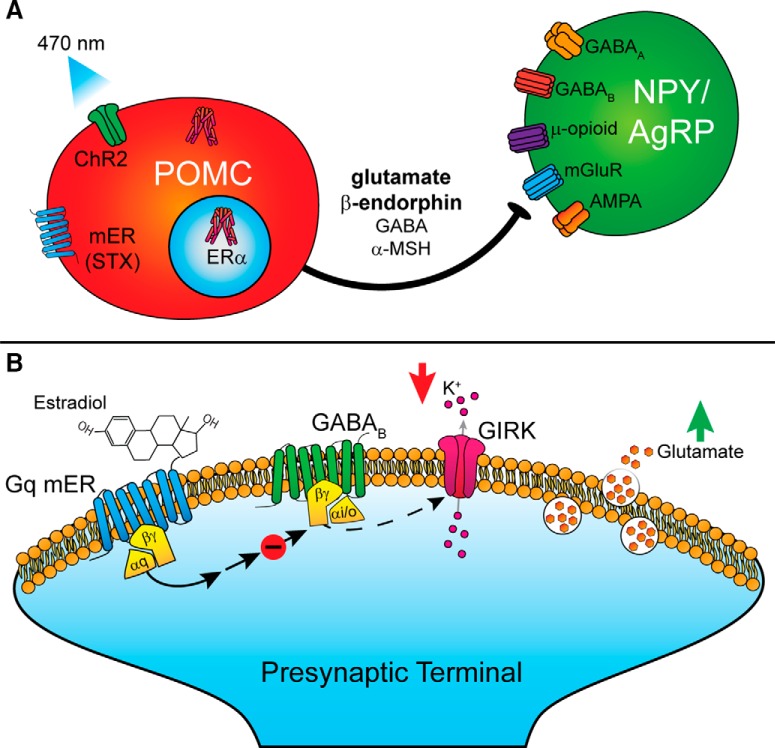
Signaling schematics. ***A***, Optogenetic stimulation of POMC neurons primarily evokes release of glutamate and β-endorphin onto NPY/AgRP neurons. These findings suggest release is segregated such that GABA and α-MSH is released on to other neuronal subtypes. Acute and chronic activation of ERs increases the release probability of glutamate from POMC onto NPY/AgRP neurons. ***B***, Activation of a Gq-coupled membrane-bound ER leads to the desensitization of GABA_B_ receptors. Decreased coupling between GABA_B_ and GIRK channels results in greater glutamate release from the terminal.

Although chronic E2 enhanced glutamate release through increased *Vglut2* mRNA expression changes, acute activation of mERs induced a similar effect (Fig. 5). Bath application of E2 and STX decreased the PPR within minutes, even when the slice was pre-treated with cycloheximide to inhibit protein synthesis. GABA_B_ receptors can reduce the release probability by decreasing presynaptic Ca^2+^ (Wu and Saggau, 1995; Dittman and Regehr, 1996), activating K^+^ channels or lowering cyclic AMP concentrations (Thompson and Gähwiler, 1992). Normally, selective activation of either GABA_B_ or opioid receptors causes a robust membrane hyperpolarization and cessation of action potential firing in ARH neurons including POMC neurons (Slugg et al., 2003; Kelly et al., 1990; Loose and Kelly, 1990; Pennock and Hentges, 2011). Therefore, this rapid E2/STX effect is likely the result of a G_q_-coupled membrane ER rapidly desensitizing GABA_B_ receptors in POMC neurons thus uncoupling it from activation of GIRK channels (Qiu et al., 2003, 2006; Fig. 8*B*) and enhancing POMC neurotransmission. This more rapid effect of E2 may help defend the set point against minor fluctuations in nutrients until changes in gene transcription are manifested.

Therefore, there are multiple signaling cascades engaged by E2 in hypothalamic neurons that coordinate reproduction and homeostatic processes, such as feeding behavior (Sinchak and Wagner, 2012; Kelly and Rønnekleiv, 2015; Micevych et al., 2017; Rivera and Stincic, 2018). E2 exerts potent anorectic effects through activation of POMC neurons (Kelly et al., 1992; Lagrange et al., 1994; Qiu et al., 2003, 2006) and inhibition of NPY/AgRP neurons (Roepke et al., 2011a; Smith et al., 2014), actions mediated through both membrane-initiated and nuclear signaling mechanisms. Specifically deleting ERα in POMC neurons is sufficient to disrupt energy balance, resulting in hyperphagia and weight gain in rodents (Xu et al., 2011). Compared to oil-treated OVX female guinea pigs, rats and non-human primates, E2 upregulates *Pomc* mRNA and increases β-endorphin protein expression in POMC neurons (Thornton et al., 1994; Bethea and Widmann, 1996; Roepke et al., 2008), whereas E2 attenuates the orexigenic actions of NPY/AgRP neurons and suppresses its expression (Crowley et al., 1985; Pelletier et al., 2007; Santollo and Eckel, 2008).

The function of POMC neurons may be to monitor external (food and mate availability) and internal (energy balance and fertility) states, shifting motivation to maximize reproductive fitness (Storm and Tecott, 2005; Shadmehr et al., 2016; Fischer and O’Connell, 2017). Therefore, constant, low firing activity of POMC neurons may be insufficient or inappropriate to engage both amino acid and peptide transmission to quickly decrease food intake. Our results would suggest that high-frequency activity of POMC neurons releases β-endorphin to provide an inhibition of NPY/AgRP activity that signals a repletion of energy stores, resetting the Flip-Flop circuit (Yang et al., 2011). In addition, we believe that our findings at this synapse will generalize to other POMC projections with differences in the expression of postsynaptic receptors and/or segregated release of neurotransmitter (Vaaga et al., 2014) determining whether the downstream target neurons are excited or inhibited. Moreover, the present findings of steroid regulation of *Vglut2* mRNA expression indicate that E2 is critical for enhanced glutamatergic and peptidergic (inhibitory) signaling from POMC to NPY/AgRP neurons in the female mouse. Certainly, elucidation of this novel peptidergic (opioid) and amino acid (glutamatergic) input to NPY/AgRP neurons from POMC neurons will help to define this complex circuitry responsible for regulating energy metabolism and homeostasis.

Acknowledgements: We thank Dr. Richard Palmiter and Dr. Stephanie Padilla at the University of Washington-Seattle for providing the viral vectors used in these experiments. We also thank Uyen-Vy Navarro, Ashley Connors, and J. G. Bradner for technical assistance and Dr. Jian Qiu for his helpful comments on the manuscript.

## Synthesis

Reviewing Editor: Quentin Pittman, University of Calgary Decisions are customarily a result of the Reviewing Editor and the peer reviewers coming together and discussing their recommendations until a consensus is reached. When revisions are invited, a fact-based synthesis statement explaining their decision and outlining what is needed to prepare a revision will be listed below. The following reviewer(s) agreed to reveal their identity: Yong Xu.

Both reviewers agree that you present interesting and good quality data. I have carefully gone through the manuscript myself and have also had extensive consultations with the reviewers concerning your manuscript. As a personal comment, I (and the reviewers) have difficulty discerning your message here- is it POMC-NPY connectivity? Is it E2 effects on transmission? Is it amino acid vs peptidergic transmission? In your revision this could be made clearer. The evidence for a peptidergic synapse is quite interesting and adds to the literature concerning the relative effects of different stimulation paradigms on release of different transmitters. Evidence of a POMC-NPY/AGRP connectivity is an important advance and adds to our understanding of possible mechanisms of satiety and food intake.

Where both reviewers agree, and I concur, is on the following points that are critical they be addressed for manuscript acceptance:

1. You make the point for a monosynaptic relation between POMC and NPY/AGRP neurons, which is indeed novel data. Your electrophysiological data ( 4AP/TEA/TTX) do support this, but the immunohistochemical figure is anecdotal , at best. First of all, how representative is this of other cells? Some quantification is necessary here- how many NPY/AGRP cells showed such proximal labelling? How many did not? Secondly, in the absence of EM, your assertion of a monosynaptic input should be more nuanced .

2. You frequently posit an mGluR receptor in the pathway ( and include this assertion in the abstract) , but show no evidence it exists. We all feel that you should at least a) demonstrate the presence of mGluRs on NPY/AGRP neurons using readily available agonists, b) report effects of mGluR antagonists on synaptic responses.

3. So where is GABA in all of this? This has been lost in the presentation and it has not properly been tested or discussed, yet played an important part in the rationale leading up to these experiments.

4. There was concern that you are using a model with likely ectopic expression of eGFP with one that you show is more selective. One reviewer suggests you should limit your data to the more specific one ( this you are may want to rebut or change data presentation). Furthermore,, in your optogenetic study you recorded from both eGFP expressing neurons and in surrounding ones and then used post hoc PCR to identify if the neurons were indeed NPY/AGRP in nature. However you do not provide information as to how many were NPY/AGRP in nature and how the responses parsed into each group.

5. There was considerable discussion whether your conclusion that vGLut levels are increased as a result of E2 is robust. You compared proestrous females with OVX females; the appropriate experiments would be to compare OVX females in the presence and absence of estrogen. It is also uncertain how increased expression of transporter levels might relate to release. Please address both of these points.

In addition , there were a number of other minor points noted by me or the reviewers:

6. The “unpublished” data regarding Oprm1 expression in single NPY neurons - either give us the data or remove.

7. Figure 5A only had N=2. More repeats are certainly needed if the authors want to include this data.

8. Figure 7B is too speculative. There is no evidence provided in the current study to show that ERalpha can directly regulate Vglut2 expression as presented in this model.

9. Figure 7C showed “Gq mER” as a membrane receptor for E2 with 7 transmembrane domains. This reviewer went back to check the cited literature (Qiu 2003, and Qiu 2006), but could not find convincing evidence that effects induced by the STX compound are mediated by a “novel membrane-associated estrogen receptor”. Has this receptor been cloned? What is the gene encoding this receptor? Is this really a membrane protein with 7 transmembrane domains, like a typical GPCR? (note from editor- one reviewer did not feel this was an issue, but you should definitely buttress this with other evidence)

9. English issue: “similar to what we has been described for kisspeptin”

As an editorial comment, neither your title nor the abstract indicates the species- please address this in a revision. Also , for replication, please indicate if the animals were held in a SPF ( specified pathogen free ) facility.

One of the reviewers provided editorial comments directly on your manuscript and this will be sent to you in a separate email.
